# Effect of pre-infusion of hypertonic saline on postoperative delirium in geriatric patients undergoing shoulder arthroscopy: a randomized controlled trial

**DOI:** 10.1186/s12871-023-02340-5

**Published:** 2023-12-11

**Authors:** Fang Xu, Yanan Li, Xupeng Wang, Ran Sun, Zilei Zheng, Qi Zhang, Mingyang Gao, Wei Tao, Juan Zhao, Qiujun Wang

**Affiliations:** 1 Department of Anesthesiology, the Third Hospital of Hebei Medical University, Shijiazhuang, Hebei China; 2 Department of Joint Surgery, the Third Hospital of Hebei Medical University, Shijiazhuang, Hebei China; 3 Department of Anesthesiology, Zhangjiakou Fourth Hospital, Zhangjiakou, Hebei China; 4 Department of Anesthesiology, Children’s Hospital of Hebei Province Affiliated to Hebei Medical University, Shijiazhuang, China; 5 Department of Breast Surgery, the First Affiliated Hospital of Jinzhou Medical University, Jinzhou, China; 6 Teaching Experiment Center, Hebei Medical University, Shijiazhuang, Hebei China

**Keywords:** Postoperative delirium, Neuroinflammation, Hypertonic saline, Neutrophils, Cytokines

## Abstract

**Background:**

Neuroinflammation may be a potential mechanism of postoperative delirium (POD) in geriatric patients, and hypertonic saline (HS) has immunomodulatory properties. The purpose of this study was to investigate whether HS could reduce the incidence of POD in elderly patients and its effect on neutrophil activation and inflammatory cytokine expression.

**Methods:**

We studied the effect of pre-infusion of 4 mL/kg 3% hypertonic saline vs. 4 mL/kg 0.9% normal saline on POD in patients undergoing shoulder arthroscopy in a prospective, randomized, double-blind, controlled trial. Neutrophil surface molecules (CD11b, CD66b and CD64) were analyzed by flow cytometry. Circulating concentrations of inflammatory factors IL-1β, IL-6, TNF-α and neurological damage factor S100β were assessed by enzyme immunoassay. The Confusion Assessment Method-Chinese Revision (CAM-CR) was applied for the assessment of POD 1–3 days after surgery.

**Results:**

The incidence of POD in group H was significantly lower than that in group N (7.14% vs 26.83%, *P* = 0.036). The expression levels of inflammatory cytokines ( IL-6 and TNF-α) and neutrophil surface markers (CD11b and CD66b) were significantly lower in group H than in group N at 24 h after surgery (*P* = 0.018, *P* < 0.001, *P* < 0.001, *P* = 0.024). There were no significant differences in postoperative pain, nausea and vomiting, infection, phlebitis, and patients satisfaction between the two groups.

**Conclusion:**

Pre-infusion of HS can reduce the incidence of POD and the immune-inflammatory response.

**Trial registration:**

Chinese Clinical Trial Registry (14/4/2022, registration number: ChiCTR2200058681.

## Introduction

Delirium is a dysfunction of attention, thought and consciousness accompanied by changes in cognitive or perceptual impairment. Patients often have an acute onset and the condition is fluctuating [1]. Postoperative delirium (POD) is one of the most common comorbidities after surgery in hospitalized geriatric patients. According to statistics, POD has been estimated to occur in 3%-50% of elderly patients after different anesthesia procedures and is directly related to morbidity, mortality, length of hospital stay and hospitalization costs [2]. Several studies have demonstrated the importance of preventing delirium in patients undergoing surgery, and the treatment of delirium has focused on both nonpharmacologic (early mobilization, limiting the use of sedative drugs and methods to improve the quality of sleep) and pharmacologic therapies (haloperidol, ketamine, melatonin, ramelteon, etc.), although the effectiveness of pharmacologic treatments has been controversial [3, 4]. A systematic review has shown that the incidence of delirium after orthopaedic surgery is 4.5–41.2% [5]. Shoulder arthroscopy is one of the most common procedures performed on the shoulder. During the procedure, large amounts of fluid irrigation and controlled hypotension are required to improve intra-articular visualization of the shoulder. Studies have reported intraoperative cerebral hypoperfusion and postoperative cognitive decline in patients undergoing shoulder arthroscopy under general anesthesia [6]. Besides the mechanisms, risk factors, clinical onset, and prognosis of POD have been studied clinically and preclinically, the specific mechanisms behind the complex clinical symptoms are unclear, and effective methods to reverse POD are still lacking.

Neuroinflammation is a possible pathophysiological mechanism of POD. Stress reactions caused by trauma, surgery and anesthesia often activate the immune system non-specifically and are characterized by increased neutrophil counts [7]. This activation may involve disruption of the blood–brain barrier, activation of microglia, and release of cytokines, which may help explain the pathophysiological mechanisms of POD [8]. As the percentage of neutrophils increases in the elderly, the inflammatory properties of circulating neutrophils change. Neutrophil phenotype may be associated with decreased cognitive function [9]. CD11b, CD66b and CD64, markers of neutrophil activation, are involved in neutrophil activation, adhesion and phagocytosis [10]. Researchers confirmed that neutrophils can produce cytokines including TNF-α, IL-1β and IL-6, and that three cytokines are involved in the inflammatory cascade response, which is correlated with a high risk of POD [11].

In addition to improving hemodynamic parameters, hypertonic saline (HS) has potential immunomodulatory effects to modulate local and systemic inflammatory responses [1]. HS reduces neutrophil activation, adhesion and degranulation, and decreases cytokine expression [12]. The aim of this research was to determine the effect of pre-infusion of HS on POD in geriatric patients undergoing shoulder arthroscopy and to initially investigate the correlation between HS and neuroinflammation and POD. We hypothesize that pre-infusion of HS can reduce the incidence of POD in elderly patients by a mechanism that may be related to reduced activation of neutrophils.

## Methods

### Study participants

The Ethics Committee of the Third Hospital of Hebei Medical University approved this study in June 2022 (No.2022-035-1). The study was registered with the Chinese Clinical Trial Registry (ChiCTR2200058681) and was conducted from June 2022 to March 2023. After written informed consent was obtained, patients planned to receive unilateral shoulder arthroscopy were included in this research. Inclusion criteria: ASA class I-III, ≥ 60 years old and general anesthesia. Exclusion criteria: History of central nervous system disease (transient ischemic attack, stroke, cerebral hemorrhage, syncope, spinal cord injury, upright hypotension), electrolyte disturbance (Hypernatremia, hypokalemia and hyperchloremia), MMSE score < 24, history of neurological or psychiatric disorders, present application of sedatives or antidepressants, infection or chronic inflammation, recent application of anti-inflammatory drugs (NSAIDs, Coxibs and more), non-consent to experimental procedures, speech impairment, illiteracy, hearing impairment or visual disorder, alcohol or drug addiction, and anesthetic drug allergy. Patients were randomly allocated to either hypertonic saline group (3% HS) or normal saline group (0.9% NS) in a 1:1 ratio via a computerized randomization table. Patients and anesthesiologists were unaware of the assignment of the two groups.

### Anesthetic management

The evening prior to surgery, MMSE cognitive test and weight measurement were performed. Patients with preoperative pain took tramadol hydrochloride sustained -release tablets for symptomatic relief. After the patient entered the operating room, the anesthesiologist assistant opened a sequentially numbered, sealed, opaque envelope containing the patient’s assignment. Patients were continuously monitored for invasive blood pressure, pulse oxygen saturation, electrocardiogram, bispectral index (BIS), and local cerebral oxygen saturation (rSO_2_, INVOS 5100TM, Somanetics Corp, USA). Patients were given an intravenous infusion of 4 mL/kg 0.9% NS or 3% HS (Shijiazhuang No.4 Phamaceutical) 30 min before anesthesia. Patients received continuous oxygen inhalation 2 L/min through a face mask. Preoperatively, the anesthesiologist conducted an ultrasound-guided brachial plexus nerve block of 30 mL of 0.5% ropivacaine on the affected side. General anesthesia was performed according to the standard protocol. The induction of general anesthesia in patients was administered with propofol 2 mg/kg, sufentanil 0.2 μg/kg, midazolam 0.1 mg/kg, and cis-atracurium 0.15 mg/kg. After tracheal intubation, ventilation was controlled mechanically, and intraoperative respiratory parameters were regulated to maintain ETCO_2_ between 35–45 mmHg. Intraoperatively, propofol (3–8 mg/kg/h) and remifentanil (0.1–0.2 μg/kg/min) were given by intravenous infusion pump, and the appropriate depth of anesthesia was maintained (BIS value was maintained at 40–60).

To minimize intraoperative blood loss, the patient’s mean arterial pressure was controlled at about 60–65 mmHg by inhalation of sevoflurane, or by intravenous infusion of esmolol or nitroglycerin, while the oxygen supply to vital organs was ensured. If the mean arterial pressure falls below 60 mmHg during surgery, dopamine or norepinephrine is given. Intraoperative cerebral desaturation events (CDEs) were recorded (rSO_2_ absolute value < 55% or ≥ 20% decrease from baseline value for ≥ 15 s), and if it occurs, the anesthesiologist gives appropriate interventions: adjustment of blood pressure and depth of anesthesia, increase of FiO_2_ or ETCO_2_. Good tissue perfusion was kept by colloid/crystal infusion. Postoperatively, the tracheal tube was taken out when the patient was able to open his eyes as instructed and maintain adequate voluntary breathing. The patient was taken to the post anesthesia care unit (PACU) for observation. Following surgery, the patient was administered intravenous patient controlled analgesia (PCA), including sufentanil (1.5 mg/kg) and ramosetron hydrochloride (0.3 mg). We set the pump’s basal flow rate to 2 mL per hour and the bolus dose to 0.5 mL. The lock-out time was 15 min. Patients were instructed to press the analgesic pump for self-administered analgesia when the VAS score was higher than 4. The patient was given tramadol hydrochloride (50 mg) intramuscular (up to 400 mg/day) if analgesia was insufficient (VAS 5–7/10). The anesthesia assistant recorded the patients’ demographics, including age, gender, height, weight, ASA classification and past medical history. The duration of intraoperative anesthesia and postoperative anesthesia-related complications were recorded. Blood gas analysis was performed before operation, 1 h after operation and 24 h after operation. Return visits were performed by investigators who were unaware of the patient subgroup and had received training prior to the study, and the CAM-CR scale was applied twice daily for 1–3 days postoperatively (from 8:00 to 10:00 am and from 18:00 to 20:00 pm) for POD assessment, a method that has been validated in Chinese patients [13].

The CAM-CR score includes: acute onset, attentional disturbance, disturbed thinking, altered level of awareness, disorientation, memory disturbance, perceptual impairment, psychomotor arousal, psychomotor retardation, fluctuation, and alteration of sleep–wake cycle in a total of 11 items, and each index was scored from 1 to 4. Each index was classified according to the severity as: 1 point: none; 2 points: mild; 3 points: moderate; 4 points: severe. The total score is 11 to 44, with 22 points as the threshold for delirium, and more than 22 points were diagnosed as delirium.

### Blood sample collection and sample measurement

Before and 24 h after the procedure, 2 mL of venous blood was drawn, placed in sterile EDTA tubes for flow cytometry analysis and cytokine testing. 100 uL of blood was added with 5 uL each of fluorescein antibodies PE anti-human CD11b (Cat No. 301306, Lot No. B338581, Biolegend, USA), PE/Cyanine7 anti-human CD66b (Cat No. 305116, Lot No. B345422, Biolegend, USA) and FITC anti-human CD64 (Cat No. 399506, Lot No. B357054, Biolegend, USA), and another 100uL of blood was added with antibodies as a control group. Specimens were incubated for 30 min at room temperature, protected from light, and erythrocytes were lysed using EPICS hemolysis instrument (Beckman Coulter Company, USA). Flow cytometry (Beckman Coulter FC 500MPL, USA) was used for detection and analysis.

The remaining venous blood was centrifuged at 3000 g for 30 min at 4 °C, and then the plasma was collected. Plasma was equated into polypropylene tubes and stored in a refrigerator at -80 °C for cytokine detection. Plasma concentrations of the inflammatory cytokines IL-1β (Cat No. CHE0001, Lot No. 20220920, Beijing 4A Biotech Co., Ltd, China), IL-6 (Cat No. CHE0009, Lot No. 20221008, Beijing 4A Biotech Co., Ltd, China), TNF-α (Cat No. CHE0019, Lot No. 20220920, Beijing 4A Biotech Co., Ltd, China) and S100β (Cat No. E-EL-H1297c, Lot No. AK03886L3479, elabscience, China) were measured using commercial enzyme-linked immunosorbent assay (ELISA) kits following the manufacturer’s instructions. ELISA test was read by URIT-660 Microplate Reader and the testers were unaware of the clinical information.

### Sample size estimation and statistical analysis

In a pretest of 12 patients per group, 1 patient in the hypertonic saline group developed POD and 4 patients in the normal saline group developed POD. The incidence of POD in the two groups was 0.083% and 0.333%, respectively. To detect a clinically significant decrease in the incidence of POD at an α risk of 0.05 (two-tailed) and a power of 80%, we needed a sample size of 38 patients per group (PASS 15.0, CNSS, UT, USA). Based on a dropout rate of approximately 10%, 42 patients were recruited in each group.

Data from the trial were analyzed by SPSS 25.0 software. Categorical variables were analyzed by chi-square test, Yates’ continuity corrected Chi-square test or Fisher’s exact test and expressed as numbers and percentages. Student’s *t*-test was applied in the case of normally distributed continuous variables, and Mann–Whitney *U*-test was applied in the case of skewed continuous variables, expressed as mean ± standard deviation.

## Results

**Fig. 1:**
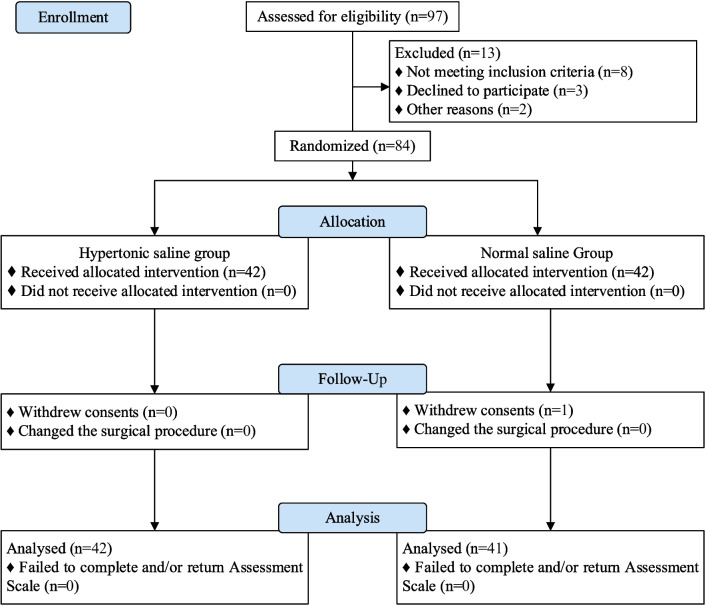
Flow diagram of study participants

Eighty-four elderly patients who underwent shoulder arthroscopy were included in this study and randomized into two groups, of which Eighty-three patients completed the trial (Fig. 1). Baseline characteristics of the patients were shown in Table 1, including demographic variables and medical history of disease. There were no statistically significant differences in gender, age, height, weight and medical history of disease between the two groups of patients (*P* > 0.05).

**Table 1:** Patients’ demographic characteristics, anesthesia time and incidence of POD

Variable	H (*n* = 42)	N (*n* = 41)	*P* value
Demographic and clinical characteristics			
Age (years) mean ± SD	65.88 ± 3.63	65.41 ± 4.26	0.593
Sex (Female) n (%)	27 (64.29%)	26 (63.41%)	0.934
Height (cm) mean ± SD	163.80 ± 7.55	163.00 ± 6.91	0.601
Weight (kg) mean ± SD	67.38 ± 10.29	67.21 ± 11.40	0.942
Body mass index (kg/m^2^) mean ± SD	25.07 ± 2.97	25.22 ± 3.38	0.828
ASA score of 2, n (%)	40 (95.24%)	38 (92.68%)	0.978
Frequency of comorbidities			
Hypertension, n (%)	15 (35.71%)	13 (31.71%)	0.700
Diabetes, n (%)	10 (23.81%)	6 (14.63%)	0.289
Coronary artery disease, n (%)	6 (14.29%)	8 (19.51%)	0.525
Serum sodium (mmol/L) mean ± SD			
Pre-operation	138.90 ± 5.78	139.40 ± 5.75	0.661
1 h after the operation	147.10 ± 2.44	141.00 ± 4.12	< 0.001^**^
24 h after the operation	140.00 ± 5.29	138.00 ± 4.28	0.069
Duration of anesthesia (min) mean ± SD	228.50 ± 53.63	231.00 ± 39.47	0.805
Cerebral desaturation events, n (%)	6 (14.29%)	14 (34.15%)	0.034^*^
Incidence of POD, n (%)	3 (7.14%)	11 (26.83%)	0.036^*^
Need for vasopressors, n (%)	8 (19.05%)	15 (36.59%)	0.074
Amount of intraoperative fluids (mL) median (IQR)	1300 (1200–1563)	1500 (1300–1600)	0.052

Values are presented as number of patients (%) or mean ± SD

*ASA* American Society of Anesthesia, *POD* Postoperative delirium

The difference between the two groups is significant, *P < 0.05, **P < 0.01

The incidence of POD and intraoperative CDEs were significantly lower in Group H compared with Group N (7.14% vs 26.83%, *P* = 0.036) (14.29% vs 34.15%, *P* = 0.034) (Table 1). There was no statistically significant difference in the use of vasopressors and the amount of intraoperative fluids between the two groups (*P* ≥ 0.05). Preoperative expression of inflammatory cytokines and neutrophil surface markers did not significantly differ between the two groups (*P* ≥ 0.05). Group H showed a significantly lower plasma IL-6 and TNF-α level than group N at 24 h after the operation (*P* = 0.018, *P* < 0.001). Additionally, group H showed a significantly lower neutrophil CD11b and CD66b expression level than group N at 24 h postoperatively (*P* < 0.001, *P* = 0.024) (Table 2).

**Table 2:** Expression of inflammatory cytokines and neutrophil surface markers

	H (*n* = 42)	N (*n* = 41)	*P* value
Cytokines (pg/mL) mean ± SD			
TNF-α Pre-operation	5.95 ± 3.35	6.91 ± 3.18	0.184
24 h after operation	16.17 ± 3.76	31.94 ± 6.47	< 0.001^**^
IL-1β Pre-operation	14.41 ± 7.48	13.66 ± 6.54	0.628
24 h after operation	19.72 ± 5.48	21.74 ± 5.43	0.096
IL-6 Pre-operation	9.87 ± 4.96	11.68 ± 6.22	0.146
24 h after operation	62.55 ± 16.60	72.83 ± 21.75	0.018^*^
S100β Pre-operation	40.68 ± 6.32	38.87 ± 8.87	0.285
24 h after operation	86.86 ± 20.22	97.33 ± 24.14	0.035^*^
Neutrophil surface markers (MFI) mean ± SD			
Neutrophil CD11b Pre-operation	6.99 ± 2.43	7.92 ± 3.12	0.134
24 h after operation	11.69 ± 4.31	21.56 ± 7.65	< 0.001^**^
Neutrophil CD66b Pre-operation	3.33 ± 1.86	3.57 ± 2.44	0.622
24 h after operation	9.02 ± 2.57	10.18 ± 1.95	0.024^*^
Neutrophil CD64 Pre-operation	1.20 ± 0.26	1.17 ± 0.26	0.542
24 h after operation	1.26 ± 0.31	1.35 ± 0.41	0.262

Values are presented as mean ± SD

*MFI* Mean fluorescent intensity

The difference between the two groups is significant, *P < 0.05, **P < 0.01

**Fig. 2:**
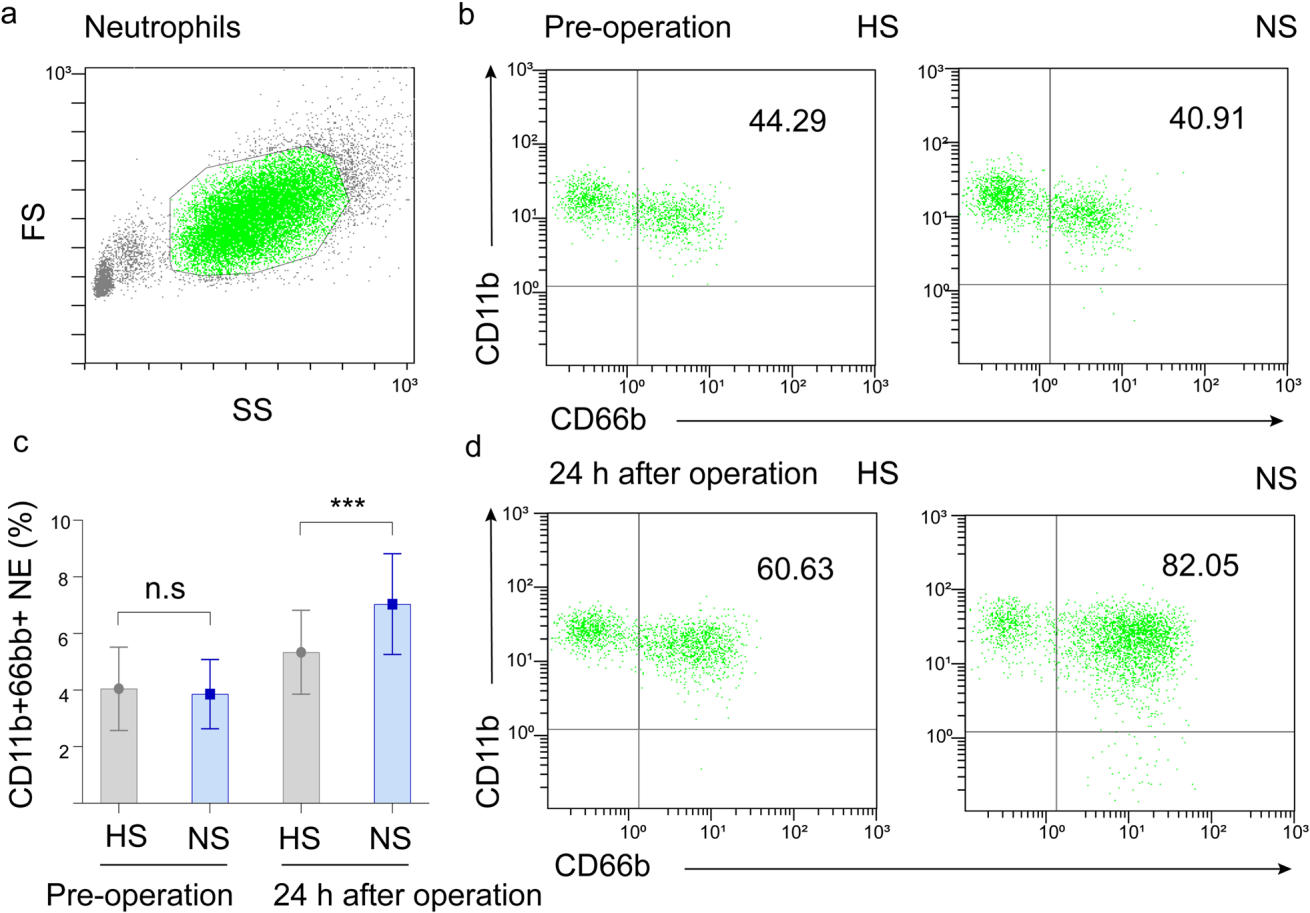
Expression of circulating neutrophil surface markers. The graph shows the percentage of CD11^+^66^+^ neutrophils analyzed by flow cytometry pre-operation (**b**) and 24 h after operation (**d**). NE: neutrophils, HS: hypertonic saline, NS: normal saline, ***P < 0.001

The percentage of CD11b^+^66b^+^ neutrophils was similar in the two groups before surgery, but was significantly lower in group H than in group N at 24 h after surgery (*P* < 0.001) (Fig. 2). There was no statistically significant difference in the amount of postoperative fluids, time to ambulation, intake of food and voiding between the two groups (*P* ≥ 0.05). Postoperative resting NRS score, postoperative nausea and vomiting, hypothermia, shivering and patients satisfaction were similar between the two groups (Table 3). No serious adverse events such as infection, phlebitis, demyelination and need for blood pressure support were observed in the two groups.

**Table 3:** Patients satisfaction and postoperative adverse events

	H (*n* = 42)	N (*n* = 41)	*P* value
Patients satisfaction, n (%)	39 (92.86%)	37 (90.24%)	0.973
PONV, n (%)	4 (9.52%)	3 (7.32%)	0.973
NRS for pain, median (IQR)			
12 h after the operation	4.00 (2.00–5.00)	4.00 (3.00–5.00)	0.446
24 h after the operation	2.00 (1.00–3.25)	2.00 (1.00–3.00)	0.252
Demyelination, n (%)	0 (0%)	0 (0%)	-
Phlebitis, n (%)	0 (0%)	0 (0%)	-
Infection, n (%)	0 (0%)	0 (0%)	-
Hypothermia, n (%)	10 (23.81%)	11 (26.83%)	0.752
Shivering, n (%)	6 (14.29%)	5 (12.20%)	0.779

Values are presented as number of patients (%), or median (inter-quartile range)

*PONV* Postoperative nausea and vomiting, *NRS* numerical rating scale, rate on a scale of 0 to 10, with higher scores indicating severer pain

## Discussion

In our study, we found that pre-infusion of HS reduced the occurrence of POD in geriatric patients after shoulder arthroscopy. The basic mechanism may be associated with the suppression of inflammatory cytokine secretion by neutrophils and improvement of cerebral perfusion. The finding that the anti-inflammatory effect of HS may be partially responsible for reducing POD confirms the neuroinflammatory hypothesis of POD.

Patients under surgery and anesthesia are at higher risk of inflammatory cascade reactions and cognitive decline compared to the preoperative steady state [8]. On the one hand, surgery can cause inflammation and blood loss; on the other hand, improper choice of anesthesia and poor intraoperative management may also lead to POD. Shoulder arthroscopy is one of the effective means of shoulder joint treatment. Because the shoulder is a special site where tourniquet cannot be applied, intra-articular fluid irrigation and Controlled hypotension are required to maintain a clear surgical field and reduce bleeding [14]. The researchers found that some patients showed significant impairment in neurobehavioral outcomes after shoulder arthroscopy due to increased neuroinflammatory markers and inadequate cerebral perfusion [15]. A significant decrease in cerebral blood flow and cerebral oxygenation was observed after the patient’s position change due to the diminished autonomic nervous system response during general anesthesia and the vasodilation caused by the anesthetic drugs [16]. Near-infrared spectroscopy technique (NIRS) can provide non-invasive, inexpensive and continuous measurements to assess cerebral oxygen saturation (rSO_2_) and brain perfusion for early detection of CDEs [17]. Previous studies have shown that CDEs are associated with cognitive decline [18]. Acute stress responses and neuroinflammation resulting from surgery, anesthesia and trauma play essential roles in the pathogenesis of POD.

POD is a common complication after surgery, especially in elderly patients. POD can cause significant short-term or long-term adverse consequences, such as loss of independence, increased morbidity and mortality [2]. Although the risk factors, clinical pathogenesis and disease development of POD have been studied, effective methods for the prevention and treatment of POD are still lacking and further research is needed. Trauma, surgery and anesthesia-induced stress responses can nonspecifically activate the immune system, characterized by increased neutrophil counts [19]. This activation may involve the release of cytokines, disruption of the blood–brain barrier, and activation of microglia [20]. Once microglia are stimulated, the central nervous system undergoes an inflammatory response followed by damage to the hippocampus, which may help explain the pathophysiology of delirium [1]. In elderly patients, microglia in the brain are more sensitive to inflammatory factors and are able to secrete more pro-inflammatory cytokines. Pro-inflammatory cytokines secreted by microglia or astrocytes can lead to cognitive decline, impaired synaptic plasticity, and altered brain function [8]. Clinical studies have shown that inflammatory cytokines in the peripheral blood of delirium patients are significantly increased in the postoperative period, demonstrating a correlation between elevated inflammatory markers in the serum or cerebrospinal fluid and the development of POD [21]. There is limited expert consensus on strategies (anesthesia selection, perioperative fluid and blood pressure management, etc.) to reduce the risk of POD. Patients would benefit from multifactorial prophylaxis and anti-inflammatory therapy if these strategies were administered prior to the onset of POD.

On the one hand, hypertonic saline (HS) improves hemodynamics, expands intravascular volume, reduces endothelial and tissue edema, decreases blood viscosity and improves microcirculation; on the other hand, HS has multiple immunomodulatory and anti-inflammatory effects, decreases neutrophil activation, transit and cytotoxicity, and reduces the release of cytokines and chemokines in plasma [22]. HS has been shown to inhibit several functions of neutrophils, including activation, adhesion, chemotaxis and degranulation [23]. In vivo and in vitro animal researches have demonstrated that central and peripheral osmotic stimulation by HS attenuates the brain’s intrinsic immune response to injury and reduces microglia and astrocyte activation, cytokine production and associated neurological damage [24]. Clinical researchers have found that patients experiencing traumatic shock trigger an inflammatory and coagulation cascade response characterized by extensive cellular activation, leukocyte and endothelial cell adhesion-induced release of pro-inflammatory cytokines and thrombus-forming mediators [19]. Administration of HS infusions to patients reduced the secretion of circulating inflammatory cytokines while increasing the concentration of the anti-inflammatory cytokines IL-1 receptor antagonist and IL-10 [25]. In our study, we found that pre-infusion of HS reduced neutrophil surface marker CD11b and CD66b, as well as inflammatory cytokines and inflammatory cascade responses. In addition, pre-infusion of HS improved cerebral perfusion and reduced the occurrence of brain desaturation events in patients.

Neutrophils are the most abundant type of leukocyte in the circulation. The half-life of neutrophils under normal conditions is approximately 12 h, while inflammation and stress can extend the life span of neutrophils to 7 days [7]. As a result of traumatic stress, surgery and anesthesia, neutrophils exhibit different phenotypes and functions that promote immune responses by releasing granules, producing cytokines and mediating the enlistment of other immune cells to the area of infection, which in turn induces an inflammatory cascade response [19]. Neutrophils are usually thought to play a beneficial role for the host, but inappropriate activation of neutrophils may also lead to autoimmune or excessive inflammatory responses, which cause tissue and organ damage. Multidisciplinary studies by immunologists and neuroscientists have concluded that there is a complex intercommunication between the nervous and immune systems and have identified a role for neuron-neutrophil communication in the pathophysiology of infection, inflammation, and neurological disease [7]. These immune cells are innovative targets for the treatment of central infections and neurodegenerative diseases. Under normal conditions, circulating neutrophils flow in the cerebral vasculature, whereas after organismal injury, inflammation triggers the expression of chemokines and adhesion molecules in the circulation, which recruit and promote neutrophil adhesion to the vessel wall. Neutrophils accumulate in the cerebral vasculature and release neurotoxic mediators, which may lead to microcirculatory obstruction and blood–brain barrier leakage [26]. Once the immune cells migrate into the brain parenchyma, they can exacerbate neurovascular damage. Scholars believe that the neutrophil phenotype is correlated with decreased cognitive function [9]. Excessive activation and degranulation of neutrophils can lead to excessive production of cytokines and chemokines, which may cause a “cytokine storm” and induce an inflammatory cascade, leading to postoperative delirium [27]. However, suppression of neutrophils may increase the risk of infection in the organism, and ideal studies need to clarify the role of neutrophils in specific neurological diseases. Researchers have worked to design specific therapies that target the harmful effects of neutrophils without compromising their beneficial protective effects [7].

CD11b, CD66b and CD64 are neutrophil activation markers that are lowly expressed in the resting state but overexpressed in response to inflammatory stimuli, and their overexpression increases neutrophil activation, adhesion, and degranulation [10]. High levels of CD11b correlated with the severity of cognitive decline in AD patients, suggesting that circulating neutrophils with the initiating phenotype may be more likely to enter the central nervous system through the cerebral vasculature [28]. CD66b is stored in neutrophil-specific granules and is mobilized to the cell surface when stimulated, participating in neutrophil adhesion and degranulation [29]. Under normal conditions, the body has low neutrophil CD64 expression. When the body is infected, neutrophil CD64 expression increases, and high levels of neutrophil CD64 expression initiate and amplify the immune response [30]. The lower mean fluorescence intensity of neutrophil CD64 in this study may be related to the low level of infection in minimally invasive surgery. In our study, we found that HS suppressed neutrophil activity and decreased inflammatory cytokine expression.

During the acute phase inflammatory response, activated neutrophils secrete large amounts of cytokines and chemokines [1]. Pro-inflammatory cytokines such as TNF-α, IL-1β and IL-6 are precise markers of the overall acute phase response, and their serum levels are used to monitor the effects of surgical trauma. Circulating pro-inflammatory cytokines initially originate in the surgical region, and TNF-α triggers the inflammatory cascade response [31]. TNF-α is not a specific marker of neutrophil activation, but it is a potent initiator of neutrophil activation and is involved in neutrophil infiltration, degranulation and oxidative responses during inflammation [32]. Studies have shown that TNF-α in the circulation is a central mediator of neuroinflammation and predicts cognitive decline in AD patients. TNF-α increases blood–brain barrier permeability and up-regulates adhesion molecule expression, which in turn exacerbates leukocyte infiltration into the brain parenchyma and leads to microcirculatory disorders [21]. IL-6 is an important cytokine linking innate and acquired immunity and is essential for peripheral and central defense against impairment and infection. Besides, IL-6 promotes IL-1β mediated inflammatory responses that cause hippocampus-dependent memory impairment. Inflammatory cytokines are considered to be a key factor in the pathogenesis of cognitive impairment and POD [33]. TNF-α produces a powerful acute effect on degenerating brain function and correlates with the severity of disease-induced brain dysfunction, including depression, delirium and postoperative cognitive dysfunction [32]. In the current research, pre-infusion of HS reduced the levels of IL-6, TNF-α and S100β compared with normal saline group, which is in accordance with prior studies that found higher levels of S100β in patients with delirium than in patients without delirium [34]. The prevalence of POD in geriatric patients undergoing shoulder arthroscopy in this study was approximately 26%. Our animal experiments have demonstrated that HS with nimodipine could improve postoperative cognitive function in elderly rats, and the fundamental mechanism may be associated with the suppression of apoptosis of hippocampal neurons [35]. Studies have demonstrated the relationship between inflammation of hippocampal neurons and postoperative cognitive dysfunction [36]. In previous clinical studies, we found that HS suppressed CD14^+^CD16^+^monocytes in geriatric patients undergoing hip replacement surgery [34]. Previous studies have focused on the application of 7.5% HS, whereas in this study 3% HS was also found to produce some anti-inflammatory effect with fewer side effects in clinical application.

The lack of preoperative assessment of blood volume status is a shortcoming of this study. Hypovolemic patients can be treated with fluid challenge therapy to increase cardiac output and optimize systemic tissue perfusion [37]. Studies have shown that the use of artificial intelligence to assess inferior vena cava variations from subcostal and trans-hepatic imaging can be used to predict fluid responsiveness [38, 39]. We will address this shortcoming and improve it in future studies. To exclude the influence of various preoperative factors, strict exclusion criteria were adopted. This resulted in the recruitment of a relatively small number of patients and limitations of the study. The observation of patients in our trial was relatively short, and prolonged observation may facilitate a more accurate analysis of the correlation between subtypes of delirium and levels of inflammatory factors. We collected plasma from patients to detect indicators of inflammation, but plasma does not adequately represent the levels of indicators in the cerebrospinal fluid, and plasma cytokine levels may increase prior to the neuroinflammatory cascade associated with delirium. Intraoperatively, we performed only cerebral oxygen saturation and BIS monitoring, and did not perform EEG monitoring, which has limitations in determining brain function.

## Conclusion

Our study verified that inflammatory and immune-related markers are elevated in peripheral blood in patients with POD. Pre-infusion of HS has a protective effect on postoperative cognitive function in elderly patients, and the mechanism of effect may be associated with the inhibition of neutrophils. Studies have shown that early control of cytokine-mediated neuroinflammation may be effective in controlling the clinical progression of POD.

## Data Availability

The datasets used and/or analyzed during the current study are available from the corresponding author on reasonable request.

## Abbreviations

ASA: American Society of Anesthesiologists
BMI: Body mass index
CAM-CR: The Confusion Assessment Method-Chinese Revision
CDEs: Cerebral desaturation events
HS: Hypertonic saline
IL-1β: Interleukin-1β
IL-6: Interleukin-6
MFI: Mean fluorescent intensity
MMSE: Mini-mental state
PACU: The post anesthesia care unit
POD: Postoperative delirium
PONV: Postoperative nausea and vomiting
rSO2: Regional cerebral oxygen saturation
TNF-α: Tumor necrosis factor alpha
